# Female genital schistosomiasis and HIV/AIDS: Reversing the neglect of girls and women

**DOI:** 10.1371/journal.pntd.0007025

**Published:** 2019-04-04

**Authors:** Peter J. Hotez, Wendy Harrison, Alan Fenwick, Amaya L. Bustinduy, Camilla Ducker, Pamela Sabina Mbabazi, Dirk Engels, Eyrun Floerecke Kjetland

**Affiliations:** 1 Texas Children’s Hospital Center for Vaccine Development, National School of Tropical Medicine, Department of Pediatrics, Baylor College of Medicine, Houston, Texas, United States of America; 2 Department of Biology, Baylor University, Waco, Texas, United States of America; 3 Schistosomiasis Control Initiative, Department of Epidemiology and Biostatistics, Imperial College London, United Kingdom; 4 Department of Clinical Research, Faculty of Infectious and Tropical Diseases, London School of Hygiene & Tropical Medicine, London, United Kingdom; 5 Uniting to Combat NTDs, London, United Kingdom; 6 Department of Control of Neglected Tropical Diseases, World Health Organization, Geneva, Switzerland; 7 Regional Advisory Unit for Imported and Tropical Diseases, Department of Infectious Diseases, Ullevaal, Oslo University Hospital, Oslo, Norway; 8 Discipline of Public Health Medicine, Nelson R Mandela School of Medicine, College of Health Sciences, University of KwaZulu-Natal, Durban, South Africa; George Washington University, UNITED STATES

Since the 2000s, we have known that female genital schistosomiasis (FGS) is likely the most neglected gynecologic condition and HIV/AIDS cofactor across sub-Saharan Africa. To date, the global health and HIV/AIDS communities have not used the opportunity to prevent new HIV/AIDS infections through highly cost-effective schistosomiasis control and elimination in Africa. But recently, this situation may be shifting toward the better.

FGS is caused by the terminal-spine parasite eggs released from the female *Schistosoma haematobium* parasite. When the eggs are deposited in the tissues of the cervix and lower female genital tract, the presence of the eggs, combined with host inflammation and increased vascularity in the cervicovaginal mucosa, produces typical intravaginal lesions that result in genital itching and pain, bleeding, and dyspareunia [1–4]. In addition, eggs deposited in the uterus and fallopian tubes can result in infertility [2, 3]. There are also associated and profound mental health effects from social stigma, such as depression and marital discord [1, 2], and the condition frequently gets confounded with sexually transmitted infections.

FGS is also incredibly common. Approximately two-thirds of Africa’s 200 million schistosomiasis cases are caused by *S*. *haematobium*, and it is estimated that up to three-quarters of girls and women with *S*. *haematobium* infection have FGS [5]. On this basis, FGS may represent sub-Saharan Africa’s most common gynecologic condition, affecting tens of millions of girls and women [6]. Yet, FGS in not mentioned in most medical textbooks, nor in the lay press, which has further compounded the very low awareness about the condition.

As if this information were not bad enough, several large epidemiological studies show that FGS is responsible for up to a three- to four-fold increase in horizontal transmission of HIV/AIDS [2, 7, 8], whereas a regression analysis of prevalence of *S*. *haematobium* infection and HIV in sub-Saharan African countries found that each *S*. *haematobium* infection per 100 individuals resulted in a 3% relative increase in HIV prevalence [9].

Given the high prevalence and incidence of FGS and its strong geographic overlap with HIV/AIDS in countries such as Malawi, Mozambique, South Africa, Tanzania, Zimbabwe, and elsewhere, it stands to reason that FGS would be identified as a leading HIV/AIDS cofactor in Africa, and that mass drug administration (MDA) with the antiparasitic drug, praziquantel, would represent an important strategy for HIV/AIDS prevention. Indeed, two transmission modeling studies found that in rural Zimbabwe, praziquantel MDA is a highly cost-effective means of reducing HIV/AIDS transmission [10, 11]. Now that praziquantel is being donated free of charge to sub-Saharan Africa by the German-based Merck KgaA for treatment of school-age children [12], praziquantel MDA may represent one of the most cost-effective means of contributing to HIV/AIDS prevention in Africa.

From the beginning of efforts to integrate MDA with praziquantel with other neglected tropical diseases (NTDs) amenable to MDA, there have been calls to link these activities with HIV/AIDS prevention efforts in Africa. Such efforts could include combining praziquantel MDA with antiretroviral treatment and pre-exposure prophylaxis (PrEP) programs, as well as other measures [13–15]. Indeed, multiple (and peer-reviewed) scientific papers have been written on this subject [13–21], but they have largely appealed to the community of scientists and public health experts committed to NTDs. Therefore, although “preaching to the converted” has helped to unify the NTDs community, it has (so far) done little to stimulate the global HIV/AIDS community toward accepting the importance of praziquantel MDA as a key component of strategies to prevent new infections of HIV/AIDS. For that reason, there was at best only modest progress on this front from the major global organizations committed to HIV/AIDS prevention, including UNAIDS, the One Campaign, the Clinton Foundation, the US President’s Emergency Plan for AIDS Relief (PEPFAR), and the Global Fund to Fight AIDS, Tuberculosis, and Malaria (GFATM). However, this situation may soon improve.

Over the last five years, the case to incorporate praziquantel MDA into HIV/AIDS treatment programs has advanced even further due to new and important developments. They include the successful testing of a new prodispersable formulation of praziquantel suitable for use in treating young (preschool age) children to prevent the onset of the genital lesions leading to FGS, together with expanded treatment programs for young children and pregnant women [20, 22–24], improved FGS diagnostic technologies and algorithms [25–31], and expanded surveillance for FGS [23, 24]. Also, there are now better basic science tools available, including a new mouse model and the applications of genomics, proteomics, metabolomics, and gene editing technologies and an expanded array of immunological reagents, to understand the pathogenesis of FGS [32–35]. Such studies could provide fundamental information on how FGS damages host tissues and leads to increased susceptibility to HIV/AIDS. Additionally, efforts have been made to develop FGS vaccines alongside HIV/AIDS vaccines, with several schistosomiasis vaccines now in clinical testing [36]. There are also efforts in place to improve local advocacy and health education around FGS [37].

Through such developments, could anti-schistosomal control efforts, including MDA, become essential elements in the HIV/AIDS prevention programmes? So far the uptake has been extremely slow. But now, both the Department of Control of NTDs at the World Health Organization, together with UNAIDS, are working towards joint programs of policy and advocacy to create some paradigm-changing shifts. Similarly, there is an urgency to integrate schistosomiasis treatments into broader health systems for women’s health, including antenatal programs, HIV/AIDS prevention programs, and cervical cancer screening clinics [23, 24, 38].

Realizing that declines in new HIV/AIDS infections remain too slow, especially in younger women aged 15–24 years who are twice as likely to be living with HIV than men [39]—in 25 countries, of which 18 in sub-Saharan Africa—UNAIDS launched its Prevention 2020 Road Map [40] calling for innovative combination prevention packages, in addition to HIV screening, counseling, and treatment programs. Since 2017, several parallel sessions have taken place at international AIDS and Women’s conferences—including the 22nd International AIDS Conference held in Amsterdam, the Netherlands in 2018—calling for a more holistic approach to women’s health and HIV. More specifically, the integration of services for HIV, FGS, Human Papilloma Virus (HPV), and cervical cancer prevention and control is called for, to improve reproductive health services and save women’s lives [41–42]. In early 2019, UNAIDS and WHO are scheduled to issue a joint Advocacy Brief on FGS and HIV.

In the meantime, the major organizations focused on integrated control and elimination of NTDs, including the WHO, continue to expand praziquantel MDA efforts in concert with the Merck KGaA donations. According to the WHO, despite making impressive gains in 2017, we are still falling short of meeting the minimum target of treating at least 75% of the African children who require regular and periodic administration of praziquantel [43]. These efforts could largely be accelerated (sustainably) were countries allowed to include them in all-out HIV/AIDS prevention and care programs supported by PEPFAR and GFATM. Incorporation with major AIDS organizations might also allow an expansion of efforts in Africa to mobilize communities, create demand for holistic female reproductive health services, and address the social stigma and mental health issues of FGS, which, for now, largely remain ignored except for a handful of one-off efforts. Additionally, medical training materials must be updated to include FGS. The clinical research community—including primary health care nurses in remote areas, pediatricians, and gynecologists—should engage in the development of appropriate treatment protocols for patients who develop FGS.

The overall neglect of the serious consequences of FGS represents an affront to the girls and women of Africa and their families in poverty-stricken communities. We have the supporting data and tools to both prevent FGS and reduce HIV/AIDS transmission in Africa. We shouldn’t continue to leave this extraordinary opportunity on the table, unused.
