# Correction: Female genital schistosomiasis and HIV/AIDS: Reversing the neglect of girls and women

**DOI:** 10.1371/journal.pntd.0008725

**Published:** 2020-09-01

**Authors:** Peter J. Hotez, Wendy Harrison, Alan Fenwick, Amaya L. Bustinduy, Camilla Ducker, Pamela Sabina Mbabazi, Dirk Engels, Eyrun Floerecke Kjetland

There is an error in the article XML causing the sixth author’s name to be indexed incorrectly. The name should be indexed as Mbabazi PS. The correct citation is: Hotez PJ, Harrison W, Fenwick A, Bustinduy AL, Ducker C, Mbabazi PS, et al. (2019) Female genital schistosomiasis and HIV/AIDS: Reversing the neglect of girls and women. PLoS Negl Trop Dis 13(4): e0007025. https://doi.org/10.1371/journal.pntd.0007025

There is an error in the article XML causing the eighth author’s name to be indexed incorrectly. The name should be indexed as Kjetland EF.
